# Supplementary material for: Efficient AI-driven allegation screening: A case study of Thailand’s National Anti-Corruption Commission
Source: PLoS One. 2026 Jan 20;21(1):e0338633. doi: 10.1371/journal.pone.0338633 (PMC12818598; doi:10.1371/journal.pone.0338633)
Supplement: Attachment — Submitted filename: Response to Reviewers.docx [file pone.0338633.s002.docx]

**Responses to Referees’ Report**

Paper Title: Efficient AI-Driven Allegation Screening: A Case Study of Thailand’s National Anti-Corruption Commission

The authors would like to thank the referees for their solid comments and suggestions. Below, please see the responses to the referees’ report. Each comment has been addressed comprehensively in a point-by-point format, with our responses presented in blue text for clarity. Additionally, all modifications made to the revised manuscript have been distinctly highlighted in red for ease of reference

| S/N | Comment | Response |
| --- | --- | --- |
|  | **Editor** | |
| 1. | Please ensure that your manuscript meets PLOS ONE's style requirements, including those for file naming | The manuscript has been revised in accordance with the PLOS ONE’s style guidelines. |
| 2. | Please provide additional details regarding participant consent. In the ethics statement in the Methods and online submission information, please ensure that you have specified (1) whether consent was informed and (2) what type you obtained (for instance, written or verbal, and if verbal, how it was documented and witnessed). If your study included minors, state whether you obtained consent from parents or guardians. If the need for consent was waived by the ethics committee, please include this information.  If you are reporting a retrospective study of medical records or archived samples, please ensure that you have discussed whether all data were fully anonymized before you accessed them and/or whether the IRB or ethics committee waived the requirement for informed consent. If patients provided informed written consent to have data from their medical records used in research, please include this information. | All participants were properly informed about the objectives and procedures of the study before providing consent. Written consent was obtained through two methods: (i) an official announcement document shared with participants, and (ii) an on-screen agreement presented on the web application interface before the commencement of the test. In addition, verbal consent was obtained and witnessed during a pre-test meeting to further ensure participants' understanding and voluntary participation. No minors were involved in this study. A waiver from the ethics committee was unnecessary, as all participants provided informed consent. This statement is provided in the methodology section (see page 17) and also has been included in the online submission system. |
| 3. | Please note that PLOS One has specific guidelines on code sharing for submissions in which author-generated code underpins the findings in the manuscript. In these cases, we expect all author-generated code to be made available without restrictions upon publication of the work. Please review our guidelines at <https://journals.plos.org/plosone/s/materials-and-software-sharing#loc-sharing-code> and ensure that your code is shared in a way that follows best practice and facilitates reproducibility and reuse. | The codes and related sources for this project can be accessed at <https://github.com/VAP-Solution/kpi_accusation_fieldtest>, which contains materials related to the field test. The test employed a custom web application developed using Appsmith, available at <https://www.vapsolution.app/nacc_llm_fieldtest>, which served as the primary interface for testing and data collection. However, the research data cannot be made publicly available due to data protection agreements with the data provider, the National Anti-Corruption Commission (NACC) of Thailand. |
| 4. | Thank you for stating the following financial disclosure: National Research Council of Thailand  Please state what role the funders took in the study. If the funders had no role, please state: "The funders had no role in study design, data collection and analysis, decision to publish, or preparation of the manuscript." If this statement is not correct you must amend it as needed. Please include this amended Role of Funder statement in your cover letter; we will change the online submission form on your behalf. | The funders had no role in study design, data collection and analysis, decision to publish, or preparation of the manuscript. |
| 5. | Thank you for stating the following in the Acknowledgments Section of your manuscript: This research was supported by the National Research Council of Thailand (NRCT), whose support enabled the research to be carried out. We also thank the National Anti-Corruption Commission (NACC) at both the central and regional levels for their collaborative efforts, data provision, and constructive feedback, which confirmed that the system met real needs. Finally, we acknowledge the unfaltering commitment and effort of our co-investigators, whose dedication was crucial in guiding the project from start to finish.  We note that you have provided funding information that is not currently declared in your Funding Statement. However, funding information should not appear in the Acknowledgments section or other areas of your manuscript. We will only publish funding information present in the Funding Statement section of the online submission form. Please remove any funding-related text from the manuscript and let us know how you would like to update your Funding Statement. Currently, your Funding Statement reads as follows: National Research Council of Thailand Please include your amended statements within your cover letter; we will change the online submission form on your behalf. | All funding-related text has been removed from the manuscript, and the acknowledgement section has been revised accordingly **Funding:** This research received funding from the National Research Council of Thailand (NRCT), Thailand |
| 6. | We note that your Data Availability Statement is currently as follows: All relevant data are within the manuscript and its Supporting Information files.  Please confirm at this time whether or not your submission contains all raw data required to replicate the results of your study. Authors must share the “minimal data set” for their submission. PLOS defines the minimal data set to consist of the data required to replicate all study findings reported in the article, as well as related metadata and methods (<https://journals.plos.org/plosone/s/data-availability#loc-minimal-data-set-definition>).  For example, authors should submit the following data:  - The values behind the means, standard deviations and other measures reported; - The values used to build graphs; - The points extracted from images for analysis.  Authors do not need to submit their entire data set if only a portion of the data was used in the reported study.  If your submission does not contain these data, please either upload them as Supporting Information files or deposit them to a stable, public repository and provide us with the relevant URLs, DOIs, or accession numbers. For a list of recommended repositories, please see <https://journals.plos.org/plosone/s/recommended-repositories>. If there are ethical or legal restrictions on sharing a de-identified data set, please explain them in detail (e.g., data contain potentially sensitive information, data are owned by a third-party organization, etc.) and who has imposed them (e.g., an ethics committee). Please also provide contact information for a data access committee, ethics committee, or other institutional body to which data requests may be sent. If data are owned by a third party, please indicate how others may request data access. | The codes and related sources for this project can be accessed at <https://github.com/VAP-Solution/kpi_accusation_fieldtest>, which contains materials related to the field test. The test employed a custom web application developed using Appsmith, available at <https://www.vapsolution.app/nacc_llm_fieldtest>, which served as the primary interface for testing and data collection. However, the research data cannot be made publicly available due to data protection agreements with the data provider, the National Anti-Corruption Commission (NACC) of Thailand. |
| 7. | If the reviewer comments include a recommendation to cite specific previously published works, please review and evaluate these publications to determine whether they are relevant and should be cited. There is no requirement to cite these works unless the editor has indicated otherwise. | No paper was recommended for citation |
| 8. | Please review your reference list to ensure that it is complete and correct. If you have cited papers that have been retracted, please include the rationale for doing so in the manuscript text, or remove these references and replace them with relevant current references. Any changes to the reference list should be mentioned in the rebuttal letter that accompanies your revised manuscript. If you need to cite a retracted article, indicate the article’s retracted status in the References list and also include a citation and full reference for the retraction notice. | During the revision process, additional relevant studies were cited to strengthen the content of the paper, particularly in the Introduction and Results sections. The newly added references are highlighted in red for ease of identification. |
|  | **Reviewer 1** | |
| 1. | Your study makes a timely and valuable contribution by demonstrating how AI can improve the efficiency of corruption allegation screening at Thailand’s National Anti-Corruption Commission, with promising results on accuracy and significant reductions in processing time; however, to strengthen the manuscript, I suggest minor revisions: the abstract could better balance methods, results, and policy implications; the introduction should more clearly outline the research gap and draw on recent comparative studies on AI in governance; the methodology would benefit from additional detail on the dataset and language-specific challenges, while the results section could be enhanced with comparative benchmarks and a brief error analysis; finally, expanding the discussion to address policy implications, potential risks such as algorithmic bias, and scalability, along with polishing a few sentences for readability, will make the paper even stronger and more impactful | The abstract has been revised to provide a balanced presentation of the methods, results, and policy implications (see pages 1-2). The introduction has been thoroughly updated to clearly articulate the research gap and incorporate comparative studies on the use of AI in governance (see pages 3-5). The methodology section has been carefully expanded to include additional details on the dataset and language-specific challenges (see pages 12-17). The results section now presents a more detailed comparison of findings, while the explanation of the error analysis has been refined for greater clarity (see pages 24-26). Furthermore, the discussion section has been comprehensively revised to address policy implications, potential risks, and scalability (see pages 32-34). Overall, the entire manuscript has undergone extensive revision to improve clarity, coherence, and conciseness. |
|  | **Reviewer 2** | |
| 1. | The publication entitled "Efficient AI-Driven Allegation Screening: A Case Study of Thailand’s National Anti-Corruption Commission" offers a pertinent and insightful examination of the integration of artificial intelligence into anti-corruption processes. The case study methodology is well implemented, providing both pragmatic insights and scholarly significance. The emphasis on Thailand’s National Anti-Corruption Commission provides a significant regional viewpoint that is frequently overlooked in AI governance research.  The study exhibits methodological rigor, good organization, and a robust understanding of AI systems and institutional settings. The discourse on screening efficiency, data prioritizing, and algorithmic openness is exceptionally well-expressed. Furthermore, the study examines critical ethical and operational issues without exaggerating the potential of AI, so fostering a fair and realistic evaluation.  The manuscript is complete and requires no modifications. The writing is lucid and professional, the figures and tables are helpful, and the references are current and relevant.  This is a significant addition to the domains of AI in governance and anti-corruption strategies. I endorse the text for acceptance in its current form, requiring only little copyediting for language consistency. | The entire manuscript has been carefully revised and thoroughly proofread to ensure consistency and clarity. |
